# Clinical Risk Factors for Life-Threatening Lower Respiratory Tract Infections in Children: A Retrospective Study in an Urban City in Malaysia

**DOI:** 10.1371/journal.pone.0111162

**Published:** 2014-10-31

**Authors:** Anna Marie Nathan, Fairuz Rani, Rachel Jiun Yi Lee, Rafdzah Zaki, Caroline Westerhout, I-Ching Sam, Lucy Chai See Lum, Jessie de Bruyne

**Affiliations:** 1 Department of Paediatrics, University Malaya Medical Centre, Kuala Lumpur, Malaysia; 2 University Malaya Paediatrics and Child Health Research Group, University Malaya, Kuala Lumpur, Malaysia; 3 Department of Obstetrics and Gynaecology, Hospital Sultan Haji Ahmad Shah, Temerloh, Pahang, Malaysia; 4 Department of Medicine, Raigmore Hospital, Inverness, Scotland, United Kingdom; 5 Julius Centre University of Malaya, Department of Social & Preventive Medicine, University Malaya, Kuala Lumpur, Malaysia; 6 Department of Biomedical Imaging, University Malaya Medical Centre, Kuala Lumpur, Malaysia; 7 Department of Medical Microbiology, University Malaya, Kuala Lumpur, Malaysia; Alberta Provincial Laboratory for Public Health/University of Alberta, Canada

## Abstract

**Aim:**

Lower respiratory tract infections (LRTIs) are an important cause of morbidity and mortality, especially in low income countries. The aim of this study was to determine risk factors of life-threatening LRTIs in hospitalised children in Malaysia.

**Methods:**

This retrospective study included children aged less than 18 years admitted for LRTIs over 13 months in a tertiary referral centre in Kuala Lumpur, Malaysia. Neonates, children with asthma and those with either no or a normal chest radiograph were excluded. Life-threatening infection was defined as that needing non-invasive ventilation or admission to the paediatric intensive care unit. Routine blood investigations and nasopharyngeal secretion results (bacterial and viral) were obtained. Chest radiographs were reviewed by a designated radiologist. Environmental data (rainfall, particulate matter ≤10 µm [PM10] and air pollution index [API]) was obtained from the respective government departments.

**Results:**

Three hundred and ninety-one episodes of LRTIs were included. Viruses were implicated in 48.5% of LRTIs, with respiratory syncytial virus (RSV) being detected in 44% of viral LRTIs. Forty-six (11.8%) children had life-threatening disease and the overall mortality rate was 1.3% (5 children). RSV was detected in 26% of children with life-threatening LRTIs. In multivariate logistic regression, chronic lung disease, presenting history of apnoea and signs of hypoxia, was associated with life threatening LRTIs. Increased LRTI admissions were associated with low rainfall but not PM10 nor API. Of those on follow-up, 39% had persistent respiratory symptoms.

**Conclusion:**

One in nine children admitted with LRTI had a life-threatening LRTI. The aetiology was viral in almost half of admitted children. RSV was detected in a quarter of children with life-threatening LRTIs. Children who present with LRTIs and either have chronic lung disease, presenting history of apnoea or signs of hypoxia, should be observed carefully as the risk of deterioration to life-threatening illness is high.

## Introduction

Three million children under five die annually in South East Asia and nearly a fifth of these deaths are attributable to pneumonia [1]. The estimated incidence of pneumonia in developing countries is 0.29 per child year compared to 0.05 per child year in developed countries [1]. Pneumonia is a common cause of hospitalisation among children and a significant contributor to morbidity and mortality in both developing and developed countries [2].

When a lower respiratory tract infection (LRTI) occurs with consolidation on the chest x-ray (CXR), it is called pneumonia. However, CXRs can be “normal” early in disease [3], [4]. Hence the World Health Organisation (WHO) defines pneumonia as the presence of age-adjusted tachypnoea with fever (≥38°C) and signs of pulmonary infection, where other causes of fever have been excluded, obviating the need for CXRs, especially in resource poor countries [5].

The range of pathogens infecting children varies widely and the difficulty in determining the causative organism is compounded by the fact that differentiating between a definite bacterial and viral aetiology based on clinical and laboratory investigations is difficult [6]. To date there is no ideal test that can differentiate bacterial and viral pneumonia accurately [7].

Most children seem to recover from their pneumonia, however there are insufficient studies that rigorously follow-up and report on the final outcome in these children. Recently an excellent follow-up study by Trenholme et al found that 74% of children post-hospitalisation for a severe LRTI either had a chronic moist cough, abnormal auscultatory chest sounds or abnormal chest radiograph 1 year later [8].

Malaysia, a South East Asian nation, lies just above the equator at latitude 2°30′N and longitude 112°30′E. There are no seasons in Malaysia and the weather is generally warm and humid with the wettest month being April and the driest, June in Kuala Lumpur. The average temperature is 27°C with an average rainfall of 250 cm^3^ per year. West Malaysia faces two monsoon seasons: November to March and late May to September.

Malaysia is a newly industrialised country and hence there may be differences from both developing and developed countries with respect to prevalence of bacterial versus viral pneumonia, provision of health services and socioeconomic issues that impact on health. In Malaysia, while healthcare services (both primary and tertiary) are reasonably accessible and efficient, our diagnostic capabilities are still lagging behind our Western counterparts. Added to this, the mind set and medical care seeking practices of the people vary and traditional medicine maintains a prominent role in society.

While Malaysia shares many environmental features and cultural influences with her South East Asian neighbours, differences in socioeconomics will affect the overall health of children. Availability and effectiveness of health care may impact on risk of life-threatening pneumonia.

Currently, in Malaysia, there are limited studies looking at aetiology and outcome of LRTIs in children [9], [10]. Chan et al in 1999 reported that in young children less than 2 years old admitted with LRTIs, 22% had a virus detected, with respiratory syncytial virus (RSV) being the commonest [9]. Widespread immunisation with pneumococcal and influenza vaccines and RSV monoclonal antibodies (Palivizumab) is also not routinely practised for economic reasons. Fortunately, *Haemophilus influenzae* type b (Hib) vaccination has been incorporated into the national immunization programme since 2000. Nonetheless, children are still at high risk for severe lung infections.

Considering the possible long term sequelae on children with severe pneumonia, it is important to determine factors that predispose children to severe LRTIs with a view to preventing deterioration and instituting effective treatment, hence reducing both mortality and morbidity of disease in a country caught between rapid industrialization and tradition.

Hence the aim of this study was to determine the clinical risk factors of life-threatening LRTIs in children. We hypothesize that young age, viral aetiology and presence of co-morbidities would increase the risk for life threatening LRTIs.

## Methodology

This study took place in University Malaya Medical Centre, a 1068 bed tertiary general hospital in Kuala Lumpur, Malaysia which serves an urban population of 1.7 million. This is a retrospective study of children admitted between 1^st^ of November 2010 to 30^th^ November 2011 with lower respiratory tract infections, i.e. with a diagnosis of pneumonia, empyema or bronchiolitis. They were between the ages of 28 days to 18 years old. In our centre, we have 130 paediatric beds, which includes a 10 bedded paediatric intensive care unit (PICU). Each general ward is equipped with a 4 bedded high dependency unit (HDU) where most of the non-invasive ventilation is done. Patients with the following conditions were excluded: Infectious asthma exacerbations, multiple trigger or viral induced wheeze, hospital-acquired pneumonia (signs of LRTI 48 hours after admission), oncological problems, a normal or no chest radiograph (CXR) taken. Ethics approval was obtained from the University Malaya Medical Centre Ethics committee (MEC Ref No: 878.18). Patient information was anonymized and de-identified prior to analysis.

### Data collection

Basic demographic data, including co-morbidities, clinical history and presentation on admission, investigation results, treatment prior to and during hospital admission and outcome after discharge (if under follow-up) were extracted from the case notes. Results for investigations which included full blood count, blood cultures, C-reactive protein, nasopharyngeal secretion bacterial and viral cultures and/or immunofluorescence(IF) for common respiratory viruses (RSV, parainfluenza 1–3, adenovirus, influenza and metapneumovirus) were collected if available. The viral IF assay used was the Light Diagnostic Respiratory Panel 1 Viral Screening & Identification Kit (Millipore, Billerica, USA). *Mycoplasma pneumoniae* microparticle agglutination (Serodia Myco II kit, Fujirebio Inc., Tokyo, Japan) antibody assays were performed on the sera of patients suspected of *Mycoplasma pneumoniae*. A positive result was defined as a single antibody titre ≥1∶320 or a fourfold or greater rise in the antibody titre in paired sera obtained 2–4 weeks apart. All blood and respiratory samples were processed and analysed by our in-house laboratory. All the laboratory data were retrieved from the hospital computer data system. Environmental factors may be important in both frequency of admission as well as severity of LRTIs [11], [12]. Hence, data on daily rainfall, daily air pollution index (API) and monthly particulate matter ≤10 µm in aerodynamic diameter (PM10) for Petaling Jaya, Selangor district was obtained from the Malaysian Meteorological Department.

Case notes were also reviewed for follow-up findings, i.e. unscheduled or scheduled doctor visits since discharge and presence of respiratory symptoms.

### Clinical case definitions

Children admitted with respiratory distress (increased respiratory rate or retractions or crepitations auscultated in the chest) and an abnormal CXR were classified as having LRTIs. In this study, all CXRs were reviewed by a designated radiologist who was blinded to the clinical data of the patients. These CXRs were scored according to Khamapirad and Glezen [13].

Cases were then classified as bacterial or viral *a priori* as shown in Table 1. This was based on a modification of the Bacteria Pneumonia Score (BPS) score [14]. In the final analysis, definite and probable cases were grouped together.

**Table 1:** Case definition of children with lower respiratory tract infections who were classified as viral, bacterial or mixed aetiology (modified from the Bacteria Pneumonia Score (BPS) score).

	Definite BacterialPneumonia	Probable BacterialPneumonia	Definite ViralPneumonia	Probable ViralPneumonia	IndeterminateAetiology	MixedAetiology
**High fever** * **≥38.5°C**	*	*	+/−	-	+/−	+/−
**TWC with ANC ≥8,000×10^9^/l**	*	*	+/−	-	+/−	+/−
**Respiratory** ** **Distress**	*	*	*	*	*	*
**Abnormal CXR** †	*	*	*	*	*	*
**Positive NPS culture** ‡ **or Blood Culture or Mycoplasma** # **Serology**	*					*
**Positive virology** §			*			*

*Highest temperature documented within 24 hours of admission;

**Signs include tachypnoea, chest recessions, presence of crepitations in the lung;

† Chest radiograph: Abnormality includes presence of infiltrates (interstitial, peribronchial or fluffy), effusion and atelectasis;

‡ Positive nasopharyngeal culture: a single bacterial organism was cultured with either a pus: epithelial cell ratio of ≥10∶1 or if there were no epithelial cells with presence of pus cells seen on microscopy [28];

# Positive *Mycoplasma* result: a single antibody titre ≥1∶320 or a fourfold or greater rise in the antibody titre in paired sera obtained 2–4 weeks apart;

§ Positive virology: Positive immunofluorescence or culture for at least one of the following respiratory viruses: respiratory syncytial virus, parainfluenzae 1–3 virus, adenovirus, influenza, or metapneumovirus.

Life-threatening disease was defined by the following end points: death or need for non-invasive ventilation or admission to PICU.

Hypoxaemia was defined as oxygen saturation <92% as per the British Thoracic Guidelines on management of community-acquired pneumonia [15]. Anaemia was defined as Hb <10 g/dl. Low birth weight was defined as birth weight <2.5 kg. Prematurity was defined as gestation <37 weeks. Hyponatraemia was defined as a level <135 mmol/l. Weight was measured and classified according to the Centre for Disease Control growth charts [16].

Chronic lung disease (CLD) included children with any chronic respiratory symptoms like tachypnoea, cough and noisy breathing but excluded children with asthma, multiple trigger or viral induced wheeze.

Environmental tobacco smoke exposure was defined as presence of any smoker in the family or caregiver’s (e.g. babysitter’s) household.

### Statistical analysis

The data was analysed using IBM SPSS Statistics 20 software. Data was described using percentage, median and interquartile range (IQR). The Chi-squared test or Fisher’s exact test (where appropriate) was used to perform univariate analysis between the clinical factors and disease outcome (life-threatening LRTI). Clinical factors included in univariate analysis, were as follows: age and age groups (less than 1 year old and 1 year and above), gender, race, presence of co-morbidities like chronic lung disease, heart disease, chronic liver and gut disease), fever (continuous data), cough, coryza, vomiting, diarrhoea, signs of lethargy, dehydration, respiratory distress, presence of rhonchi, prematurity, sodium level, neutrophil level, bicarbonate level, oxygen saturation and fever at admission, birth weight <2.5 kg, current weight centile <3^rd^, attending nursery or daycare, exposure to ETS in the house, previous history of pneumonia. Multivariate analysis was performed using binary logistic regression for significant factors identified using univariate analysis. A p-value of <0.05 was considered to be statistically significant. Measure of association was presented as odds ratios (OR) with 95% confidence intervals (CI). Correlation between environment variables (monthly rainfall, monthly API and monthly PM10) and frequency of admissions of LRTI (including life-threatening LRTIs) were tested using Pearson Correlation Coefficient. Association between rainy days and poor API (>50) with admission of LRTIs were also tested using Chi-squared test.

## Results

There were 604 admissions for LRTIs in these 13 months. Children with a final diagnosis of multiple trigger or viral induced wheezing (n = 75), who had a normal (n = 41) or no chest radiograph (n = 12) and neonates (n = 14) were excluded. Another 71 case notes were irretrievable. Finally 391 children with LRTIs were included in this study.

The median (range) age of the patients was 8 (1–211) months. The majority of the patients were l year old or less (n = 344, 88%). Other demographic characteristics are shown in Table 2. There were only 6 children who were 5 years and above with LRTI. Common presenting symptoms were cough (98%), coryza (86%), fever (80%) and shortness of breath (76%). Fifteen children had chronic lung disease: CLD of prematurity (n = 10), recurrent pneumonia (n = 4) and laryngomalacia (n = 1). Aetiology of pneumonia was identified, as defined in Table 1, in 59.1% (n = 231) of children of which 48.1% (n = 188) were of viral origin. In 65 children, viral IF was not done. Mycoplasma serology was done in 9 children as this is usually done in children 5 years and above. Viruses were detected in 98 of the 188 patients classified as viral aetiology. The commonest virus was RSV which was isolated in 43.6% of children with viral LRTIs. Fifty-nine percent of children had prior antibiotics before hospital admission (108 out of 183). Faltering growth (weight less than the 3^rd^ centile for age and sex [16]) was present in 9.7% of children. Thirty-one percent of children attended day care facilities and 45% of children were exposed to environmental tobacco smoke (ETS). Nearly 15% of children had not received Hib vaccine, 7 children had received the influenza vaccine while none had received pneumococcal vaccination. The median (IQR) duration of hospitalisation was 4 (3–6) days. Ninety-three blood cultures (23.8%) were done of which only one was positive (1.1%).

**Table 2:** Demographic characteristics and clinical findings in the 391 children admitted with lower respiratory tract infections.

Patient characteristics	
Male/Female (%)	59.3/40.7
**Race (%):**	
Malay	303 (77.5)
Chinese	22 (5.6)
Indian	54 (13.8)
Others	
**Comorbidity** * **(n = 70) (%)**	
Prematurity	43 (11.0)
Cardiac disease	23 (5.9)
Genetic/metabolic	20 (5.1)
**Clinical Findings:**	
Temperature**	37.2 (36.5–38.2)
Heart rate**	150 (27.5)
Respiratory rate**	48 (38–58)
Oxygen saturation**	97 (95–99)
Lethargy (%)	62 (15.9)
Dehydration (%)	20 (5.1)
Wheezing (%)	121 (30.9)
**Laboratory results:**	
WBC**	13.6 (10.5–17.6)
Neutrophil count (x10∧9/l)**	6.2 (3.7–9.4)
Sodium (mmo/l)**	135 (134–137)
**CXR findings (n = 391)** † **:**	
Infiltrates	346
Hyperinflation only	41
Pleural effusion	45
**Aetiology of LRTI (%):**	
Bacteria‡	31 (7.9)
Viral‡	188 (48.1)
Mixed	12 (3.1)
Indeterminate	160 (40.9)
**Viruses isolated (n = 98) (%)**	
Respiratory syncytial virus	82 (21.0)
Adenovirus	7 (1.7)
Parainfluenza virus	6 (1.5)
Influenza virus	2 (0.5)
Human metapneumovirus	1 (0.3)
**Supplementary respiratory support (%)**	
Intranasal oxygen	191 (48.2)
Non-invasive ventilation	24 (6.1)
Invasive ventilation	17 (4.3)

*1 patient may have more than 1 comorbidity;

**median (IQR);

† CXR may have 2 findings hence number adds up to more than 391;

‡ includes both definite diagnosis and probable diagnosis as predefined in Table 1.

**Figure 1:**
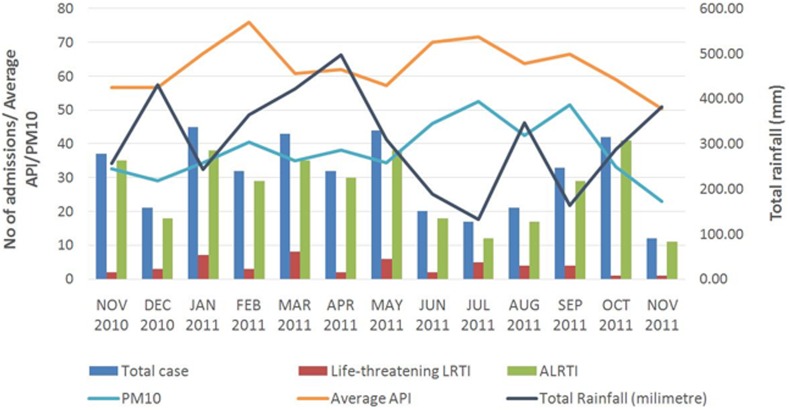
Graph showing association between total admissions for lower respiratory tract infection/month in children and rainfall/month, air pollution index [API]/month and particulate matter ≤10 µm in aerodynamic diameter (PM10)/month.

The highest rainfall was recorded in the months of March, April and December while PM10 was elevated during the months of June, July and September (Figure 1). Number of days where the API was more than 100 (unhealthy level) was eight (8) days. No significant correlation was found between monthly PM10 level (*r* = −0.13, p = 0.68), monthly API (*r* = 0.02, p = 0.96) and monthly rainfall level (*r* = 0.05, p = 0.86) with total admissions for LRTI. There was also no significant correlation was found between monthly PM10 level (*r* = 0.20, p = 0.52), monthly API (*r* = 0.22, p = 0.46) and monthly rainfall level (*r* = 0.09, p = 0.76) with admissions for life-threatening LRTIs.

Having no rain on the day was associated with increased admissions for LRTI with Odds Ratio (95% CI) = 1.77 (1.16–2.68). Unhealthy API (OR = 2.14 (0.43–10.72)) and poor API (OR = 1.09 (0.62–19)) were not found to be associated with admission for LRTI.

Fifty-nine percent of children required some respiratory support as shown in Table 2. Significant organisms isolated in nasopharyngeal secretions were *Haemophilus influenzae* (n = 20), *Staphylococcus aureus* (n = 9), *Streptococcus pneumoniae* (n = 8), *Pseudomonas aeruginosa* (*n = 4*), *Escherichia coli* (*n = 1*) and *Acinetobacter baumannii* (*n = 2*).

There were 46 cases of life-threatening LRTIs (11.8%): Forty-one children required supplementary ventilation while 5 patients required PICU care. The median (IQR) duration in PICU was 5 (3–7) days. Forty-three children (93.5%) were 12 months old or less. RSV was isolated in 26% of those who had life threatening LRTI. The overall mortality rate was 1.3% (n = 5).

In univariate analysis, seventeen factors were significantly associated with life-threatening LRTI and these were as follows: faltering growth, presence of comorbidities i.e. CHD, CLD and genetic/metabolic disease, symptoms of being afebrile, shortness of breath, apnoea and lethargy, previous lung infection, previous hospital admission, hypoxia (<92%), not attending nursery, positive NPS culture CRP, neutrophil count, lymphocyte count and bicarbonate level (Table 3).

**Table 3:** Results of univariate analysis of significant factors associated with life-threatening lower respiratory tract infections (LRTIs).

Characteristics of patients	Life-threatening LRTIs (n = 46)	Controls (n = 345)	Univariate (p value)	Crude OR (95% CI)
**Congenital heart disease**				
Yes	8	15	0.003	4.63
No	38	330		(1.84–11.64)
**Chronic lung disease**				
Yes	8	7	<0.001	10.17
No	38	338		(3.49–29.54)
**Genetic/metabolic disease**				
Yes	8	12	<0.001	5.85
No	38	333		(2.25–15.15)
**Previous pneumonia**				
Yes	11	33	0.004	2.97
No	35	312		(1.38–6.4)
**Nursery**				
Yes	7	113	0.015	0.37
No	39	232		(0.21–0.95)
**Previous admission**				
Yes	19	88	0.025	2.05
No	27	256		(1.09–3.86)
**Fever**				
Yes	28	285	0.002	0.35
No	17	60		(0.18–0.67)
**History of shortness of breath**				
Yes	42	247	0.004	5.00
No	3	88		(1.51–16.39)
**Weight**				
<3^rd^ centile	9	29	0.030	2.72
≥3^rd^ centile	36	316		(1.19–6.21)
**History of apnoea**				
Yes	6	6	<0.001	9.35
No	36	335		(2.85–30.30)
**Signs of lethargy**				
Yes	17	45	<0.001	3.91
No	29	300		(1.99–7.69)
**Signs of hypoxia**				
Yes	26	18	<0.001	23.8
No	20	327		(11.11–50.00)
**Nasopharyngeal culture**				
Positive	10	36	0.047	2.19
Negative	32	253		(1.00–4.85)
**Neutrophils (10^9^/l)** *	7.8 (6.2)	5.9 (5.4)	0.040	Z = −2.07
**Bicarbonate (mmol/l)** *	21 (5)	20 (4)	0.020	Z = −2.28
**CRP (mg/l)** *	2.8 (7.7)	1.4 (3)	0.040	Z = −2.07

*Continuous variables are represented as median (IQR).

Aetiology of pneumonia, age group, prematurity, anaemia, low birth weight (<2.5 kg), exposure to ETS, prior treatment with antibiotics, being RSV positive and hyponatraemia were not associated with life-threatening LRTIs.

In multivariate analysis, presence of chronic lung disease (OR 5.89, 95% CI 1.55, 22.43), presenting history of apnoea (OR 5.5, 95% CI 1.26, 24.13) and signs of hypoxia (OR 17.05, 95% CI 7.60, 38.22) were the only 3 independent factors associated with life-threatening LRTIs (Table 4). The Receiver Operating Characteristic (ROC) curve had an Area Under the Curve (AUC) of 0.799 (p<0.001) for these factors. This suggests that the presence of chronic lung disease, presenting history of apnoea, and hypoxia will be able to predict 79.9% of life-threatening LRTIs. Calculation of power, based on the lowest significant factor i.e. apnoea, revealed that the power of the study to detect significant factors was 97.2%.

**Table 4:** Multivariate analysis of factors that was significantly associated with life-threatening lower respiratory tract infections in children.

	Odds ratio	95% Confidence intervals	P value
**History of apnoea**	5.51	1.26–24.13	0.02
**Chronic lung disease**	5.89	1.55–22.43	0.009
**Hypoxia (<92%)**	17.05	7.60–38.22	<0.001

One hundred and sixty-five children were seen at median (IQR) of 24.5 (14–55) days post discharge and 65 children (39.4%) had respiratory symptoms.

## Discussion

We found that one in nine children who were admitted with LRTI had life-threatening disease requiring PICU or HDU care. Clinical factors associated with life-threatening disease were presence of chronic lung disease, history of apnoea and signs of hypoxia.

The Child Health Epidemiology Reference group (CHERG), an independent panel of experts particularly interested in the worldwide burden of pneumonia in children, reviewed 35 studies on various aspects of pneumonia in children, including studies from developing and developed countries [17]. They found that 11.5% of cases progress to severe disease, a finding similar to ours. Another study by Tiewsoh et al., looking at 200 children in India, found that 20.5% of children required mechanical ventilation [20]. Their mortality rate was 10.5%, much higher than in this study. This could be due to differences in baseline characteristics between the cohorts, for example in Tiewsoh’s study, 13% of children were malnourished.

While we did not find that young age was a significant factor associated with life-threatening pneumonia, this was a significant finding in a study done by Zhang et al. [18] However, in our study, young children less than 1 year old, made up more than four fifths of children admitted for LRTI. Young children, due to their relatively immature immune system and disadvantaged pulmonary mechanics, have a lower threshold for decompensation with a lung infection. However the exact pathophysiology and inflammatory mechanism is poorly understood [19].

There is extensive data on risk factors for pneumonia but not for life-threatening LRTIs requiring intensive or HDU care. Recently Zhang et al. looked at 707 children who were admitted to intensive care for very severe CAP over a 4 year period [18]. They found that young age (≤12 months old) and congenital heart diseases were significantly associated with the need for intensive care admission. They also found that children with Down syndrome and immunodeficiency had higher mortality [18]. Unlike Zhang et al.’s study, in our cohort, a diagnosis of chronic lung disease not congenital heart disease was associated with life- threatening LRTIs. This could be due to the small sample size in our study. However, there were more children with CHD than CLD (23 versus 15) included in this study. Tiewsoh et al found that cyanosis and head nodding were associated with invasive ventilation while altered sensorium, pallor, cyanosis and head nodding were associated with death [20]. In our study, signs of hypoxia were associated with life threatening disease, similar to Tiewsoh’s study. These are obviously important signs of respiratory failure.

The CHERG study found that RSV was the commonest aetiological agent identified followed by influenza. Bacterial infection, especially due to *Streptococcus pneumoniae* and *Haemophilus influenzae,* was also implicated as a significant cause of death in 33% and 16% of cases, respectively [17]. In a recently published review on organisms isolated in both children and adults in Thailand, Cambodia and Vietnam, *Streptococcus pneumonia and Haemophilus influenzae* were the commonest isolated organisms in children in Cambodia [21].

Similarly, in this study, viruses were a common aetiology among patients requiring hospitalisation with LRTIs, and RSV was isolated in 20% of admitted patients and in 26% of those who had life-threatening LRTI. Zhang et al., in a different paper but based on the original cohort mentioned above, reported that RSV was an aetiological agent in 24% of all patients with severe/very severe pneumonia, similar to our findings [22]. In another study published in 2008, the authors reported that nearly 50% of viral LRTIs in intensive care required ventilation hence emphasising the significant morbidity of viral LRTIs [23].Studies in Malaysia done by Chan et al found that RSV was responsible for 84% of cases where viruses were detected [9], [10].

Unfortunately, the yield of bacterial pneumonia in this study was very small due to the lack of use of molecular techniques in identifying bacteria. *Haemophilus influenzae* was more commonly isolated while *Streptococcus pneumoniae* was the infective agent in only 9 cases.

In Tiewsoh et al.’s study, bacteria was implicated as the cause for most disease [20].We did not find any correlation between known risk factors for LRTIs like faltering growth, anaemia, neutropenia, hyponatraemia, exposure to ETS, prematurity and metabolic acidosis. Li et al. in studying infants and young children with severe pneumonia found significant associations with metabolic abnormalities like hypocalcemia, abnormal potassium levels and metabolic acidosis [24].

The effect of certain environmental factors on lower respiratory tract infections was looked at in this study. While there was no effect of environmental factors on life-threatening LRTIs, we found a significant association between increased admissions for LRTIs and no rain. It is well known that in cold climates, increased admission for LRTIs, especially of viral aetiology, occur during winter with low temperatures [25].As for the tropics, a study done in the Philippines showed that lack of sunshine was associated with pneumonia, contrary to what we have shown [11]. Another study from Vietnam showed that increase in PM10 was associated with increased admissions for LRTIs in children with a lag effect of 1–6 days [26].

Limitations of this study are recognised. The number of life-threatening LRTIs was small. Due to the retrospective nature of this study, lack of uniform aetiology testing as well as the low use of molecular methods like polymerase chain reaction assays to determine the aetiology of pneumonia, the yield, especially for bacterial pneumonia, was low. However these molecular methods have their own limitations and confidently proving causation is still contentious [27]. We were also unable to determine other sociodemographic factors like overcrowding, indoor pollution, access to clean water and poor access to care, which could be risk factors associated with life threatening disease.

In conclusion, one in nine children hospitalised with a LRTI had a life threatening illness. RSV was isolated in more than a quarter of very ill children. Low rainfall was associated with increased admission. Factors independently associated with life-threatening disease were chronic lung disease, history of apnoea and signs of hypoxia. Healthcare staff caring for such children should be wary of these red flags in children and act fast to institute appropriate therapy.
